# On the Nitrate of Silver in Croup

**Published:** 1848-06

**Authors:** James Bryan


*On the Nitrate of Silver in Croup.* By James Bryan, M. D., etc.

To Prof. Huston.

Dear Sir,—The following case of membranous croup, treated by the application of the nitrate of silver, I send to you with the hope that some of my medical brethren will be induced to try the treatment in these confessedly mortal cases. I think it probable, that keeping the laryngeal opening patulous, until a change takes place in the disease, is of itself a great benefit, independent of the action of the salt upon the inflamed mucous membrane.

On the 21st of April, of the present year, I was called upon by my friend Dr. T. Beasly, to see with him the only child of Thomas Hutchinson, aged fourteen months, labouring under an attack of croup. From the conviction that it was a pure case of pseudo-membranous croup, little hope was expressed by Dr. B. that the child would recover. At 7 o'clock, P. M., the first application was made into the larynx, with a solution of forty grains of nitrate of silver to the ounce of water. The bent handle of a silver spoon served as a spatula to depress and draw the tongue forward. The epiglottis was distinctly seen, and the sponge cut in a conical form, and firmly fastened to a properly curved piece of whalebone, was rapidly passed behind it, and into the larynx. A temporary spasm of the glottis followed, and a free discharge of membranous and mucous fluid took place. This was succeeded by an improvement in respiration. The pulse was one hundred and thirty per minute, and thready.

9½ P. M. respiration has improved somewhat; a free discharge of mucus by vomiting had taken place since the first application. The second application was followed by a copious flow of flaky and stringy mucus, white almost as milk; some blood from the nose was mixed with the discharge; epistaxis, however, has existed now and then, ever since the disease began.

22d. 8½ A. M. The child has passed a tolerably easy night, free bilious evacuations from the bowels, the effect of two grains of calomel administered every two hours, since yesterday morning. Respiration now easy; the head is not thrown back as before; the child is in a quiet sleep; pulse ninety-five and regular; drinks cold water freely since the first application of the nitrate.

Third application, sixty grains to the ounce, into the larynx, followed by less spasm, very little irritation, and by free expectoration. Continue calomel two grains every four hours.

7 P. M. Three or four stools have been passed during the day. The child lies languidly on the pillow, with its chin raised, but quiet. The respiration dry and difficult. The first attempt at an application this evening failed, on account of the restlessness of the child, and the spasm which followed was great, and continued for several minutes. In the second attempt I succeeded in passing the instrument far down into the larynx, and brought up with it a quantity of tenacious mucus. The withdrawal of the instrument was followed immediately by the discharge of a large quantity of thick membranous, tenacious, stringy mucus, somewhat streaked and yellowish, which resulted in the complete relief of the child, who laid back his head and went to sleep in a few seconds.

23d. 8½ A. M. The respiration of our patient is comparatively easy; slept well last night; has had four bilious stools. He is so much relieved that we resolve *not* to apply the salt at present, but to hold ourselves in readiness to make the application, should it be demanded during the day.

6 o'clock P. M. The child is sitting on his mother's lap, playing with his toys. Respiration slightly stridulous; has taken bread and milk; had three stools during the day, and has slept comfortably. The throat, as far as can be seen, is free from the diptheritic deposit which at the first and second visits, had been very evident, covering the fauces and soft palate with a milk coloured membrane. Made no application this time, but directed to continue calomel one-half grain every four hours, with one grain of quinine in syrup.

24. We met again at 9¼ o'clock, A. M., and found the child lying comparatively easy in the cradle; but little sound in the respiration, which was but slightly impeded; had passed a comfortable night, slept well, taken nourishment, and passed three stools; no application; calomel to be continued; consultation to cease. Dr. Beasly informs me that the child got perfectly well, without a bad symptom, and that he thinks that the application was the means of saving its life.

It will be seen that none of the usual remedies, such as bleeding, emetics, cathartics, tobacco, &c. &c., with the exception of a few grains of calomel, were used in this case.

